# Mechanoreceptor Piezo1 Is Downregulated in Multiple Sclerosis Brain and Is Involved in the Maturation and Migration of Oligodendrocytes *in vitro*

**DOI:** 10.3389/fncel.2022.914985

**Published:** 2022-05-26

**Authors:** Maria Velasco-Estevez, Nina Koch, Ilona Klejbor, Fionä Caratis, Aleksandra Rutkowska

**Affiliations:** ^1^H12O-CNIO Hematological Malignancies Group, Clinical Research Unit, Centro Nacional de Investigaciones Oncologicas (CNIO), Madrid, Spain; ^2^Department of Laboratory Medicine, Medical University of Gdańsk, Gdańsk, Poland; ^3^Department of Anatomy, Collegium Medicum, Jan Kochanowski University in Kielce, Kielce, Poland; ^4^Department of Anatomy and Neurobiology, Medical University of Gdańsk, Gdańsk, Poland

**Keywords:** mechanoreceptor, piezo1, migration, MO3.13, oligodendrocyte (OLs), multiple sclerosis, mechanosensitive ion channel (MSC), mechanosensation

## Abstract

Mechanical properties of the brain such as intracranial pressure or stiffness of the matrix play an important role in the brain’s normal physiology and pathophysiology. The physical properties are sensed by the cells through mechanoreceptors and translated into ion currents which activate multiple biochemical cascades allowing the cells to adapt and respond to changes in their microenvironment. Piezo1 is one of the first identified mechanoreceptors. It modulates various central nervous system functions such as axonal growth or activation of astrocytes. Piezo1 signaling was also shown to play a role in the pathophysiology of Alzheimer’s disease. Here, we explore the expression of the mechanoreceptor Piezo1 in human MO3.13 oligodendrocytes and human MS/non-MS patients’ brains and investigate its putative effects on oligodendrocyte proliferation, maturation, and migration. We found that Piezo1 is expressed in human oligodendrocytes and oligodendrocyte progenitor cells in the human brain and that its inhibition with GsMTx4 leads to an increment in proliferation and migration of MO3.13 oligodendrocytes. Activation of Piezo1 with Yoda-1 induced opposite effects. Further, we observed that expression of Piezo1 decreased with MO3.13 maturation *in vitro*. Differences in expression were also observed between healthy and multiple sclerosis brains. Remarkably, the data showed significantly lower expression of Piezo1 in the white matter in multiple sclerosis brains compared to its expression in the white matter in healthy controls. There were no differences in Piezo1 expression between the white matter plaque and healthy-appearing white matter in the multiple sclerosis brain. Taken together, we here show that Piezo1-induced signaling can be used to modulate oligodendrocyte function and that it may be an important player in the pathophysiology of multiple sclerosis.

## Introduction

In the past decade, significant efforts have been made to study the biomechanical properties of the central nervous system (CNS) in health and disease. They succeeded in demonstrating that not only chemical but also mechanical cues can drive the progression of many pathophysiological states. Accordingly, several studies have revealed that oligodendrocytes and oligodendrocyte precursor cells (OPCs) are sensitive to mechanical signals. One of the very first investigations revealed that oligodendrocytes are topographically sensitive and are capable of sensing mechanical cues (Webb et al., 1995). In these experiments, synthetic substrata with regularly spaced surface contours, similar to the topographical patterns present in the CNS axons, were used to study OPC, oligodendrocyte, astrocyte, and neuronal capabilities to undergo parallel alignment. The OPCs and oligodendrocytes appeared to be the most topographically sensitive cell type of the CNS aligning on surface contours as sparse as 100 nm.

It has long been known that axon caliber determines its myelination state (Friede, 1972) and, indeed, oligodendrocytes were shown to myelinate electro-spun polystyrene nanofibers of similar diameter to the myelinated axons in an analogous way they do *in vivo* (Lee et al., 2012). Remarkably, these cells can myelinate paraformaldehyde-fixed axons demonstrating that axonal signals are not required for oligodendrocytes to begin myelination. These data indicate that, at least in the initial stages of myelination, it is the mechanical cues that are indispensable for the wrapping of myelin along the axon (Rosenberg et al., 2008). Likewise, mechanical cues seem to contribute to the differentiation of OPCs into myelinating oligodendrocytes. Research shows that OPC differentiation is inhibited in stiff substrates and by the proximity to branching and maturing oligodendrocytes, and enhanced in soft substrates (Hernandez et al., 2016; Urbanski et al., 2016; Jagielska et al., 2017). Interestingly, demyelination does not lead to a decreased stiffness of the brain (Wuerfel et al., 2010; Schregel et al., 2012), it was shown that when demyelination is chronic, as in progressive multiple sclerosis (MS), the overall stiffness of the tissue increases as a result of astrogliosis and aberrant depositions of extracellular matrix (ECM) components such as fibronectin (Urbanski et al., 2019).

The physical properties of the environment are sensed by the cells through mechanoreceptors such as Piezo1. This mechanosensitive receptor was first reported by Patapoutian’s group in 2010 (Coste et al., 2010) and has since attracted substantial attention in different aspects of normal biology and pathophysiology of diseases. Indeed, Ardem Patapoutian was awarded the Nobel prize in Physiology or Medicine in 2021 for the discovery and characterization of this mechanically sensitive ion channel.

Piezo1 is differentially expressed in the CNS at normal physiological and pathological states. For instance, this mechanoreceptor was first reported in the neuroblastoma Neuro2A cell line (Coste et al., 2010) and it has been since observed to be constitutively expressed in neurons both in the human and rodent brain (Velasco-Estevez et al., 2018; Wang et al., 2019; Roh et al., 2020). Interestingly, its expression increases in neurons during ischemia (Wang et al., 2019). In the glial cells, we were the first ones to report that Piezo1 was absent or expressed at low levels in astrocytes in normal physiological conditions. However, after a challenge with bacterial lipopolysaccharide (LPS) (Velasco-Estevez et al., 2020b) or in an aging transgenic mouse model of Alzheimer’s disease (Velasco-Estevez et al., 2018) the expression of Piezo1 increases, contributes to the inflammatory response of astrocytes and further amplifies the amyloid plaque-induced upregulation of Piezo1 in astrocytes.

Microglia express Piezo1 under normal conditions, but similarly to astrocytes, LPS challenge leads to an increment in Piezo1 expression (Liu et al., 2021). Regarding the oligodendrocyte lineage, only recently has Piezo1 expression been described in rodent OPCs (Segel et al., 2019). Piezo1 is one of the key intermediates through which OPCs sense the matrix stiffness. In stiffer substrates, OPCs stop differentiating and proliferating while inhibition of Piezo1 allows the OPCs to differentiate and proliferate in a stiff microenvironment (Segel et al., 2019). Furthermore, we showed that inhibition of Piezo1 with GsMTx4 protects from lysophosphatidylcholine (LPC)-induced demyelination in rodents, suggesting that inhibition of this mechanoreceptor could have a protective effect by lowering Ca^2+^ signaling, activating calpain, PLA_2_ and subsequent lipid degradation of the myelin (Velasco-Estevez et al., 2020a). Not only Piezo1 seems to have a direct role in the formation/degradation of myelin, but it seems to regulate the immune response associated with MS as well. It has been shown that in the mouse model of MS, the experimental autoimmune encephalomyelitis (EAE), the disease course was less severe in mice with Piezo1-deficient T cells and resulted in increased TGFβ signaling and an overall regulatory T cell (T_*reg*_) numbers (Jairaman et al., 2021). Subsequently, mice with Piezo1 knock-out T_*reg*_ cells also showed decreased EAE severity and enhanced function of T_*reg*_ cells.

Here, we explored the expression of the mechanoreceptor Piezo1 in oligodendrocytes in the human brain and its function in human MO3.13 oligodendrocytes. Our data showed that Piezo1 expression changes at different maturation stages of MO3.13 oligodendrocytes and that its inhibition induces oligodendrocyte proliferation and migration *in vitro*. Furthermore, Piezo1 was downregulated in the white matter of MS brains compared to non-MS tissue, with no differences in expression between MS plaque and healthy appearing WM within the same MS brain.

## Materials and Methods

### Ethics Statement

Human brain sections from MS and non-MS patients preserved in formalin or frozen were obtained from the Rocky Mountain MS Centre Tissue Bank (Englewood, CO, United States) after approval by the bioethics committee at the Medical University of Gdańsk, Poland (NKBBN/253/2018).

### Immunohistochemistry

For immunohistochemistry of post-mortem human brains, five MS and five non-MS coronal sections of cerebrum preserved in formalin were used. The location of the periventricular plaques varied between each patient. Visible periventricular lesions with surrounding healthy-appearing tissue were cut out (blocks of about 1 cm × 1 cm) from the brain sections and matching areas were collected from the control brains. The tissue blocks were dehydrated in 15% sucrose solution followed by dehydration in 30% solution. The tissue embedded in Tissue-Tek OCT Compound (#4583, Sakura) was cut into 30 μm slices on a cryostat and kept in antifreeze solution at −20°C until needed. Prior to staining, the sections were washed 3 times with PBS, submerged in a citrate buffer (pH 6) and microwaved at 800 W, then cooled down to room temperature on ice and microwaved again. The cooled down sections were washed twice for 2 min in PBS, then once for 10 min and finally permeabilized twice for 10 min in gelatin supplemented with 0.25% Triton-X in PBS. Then the sections were incubated in the blocking buffer [5% bovine serum albumin (BSA) in the permeabilization solution] for 2 h and then incubated overnight (o/n) with primary antibodies diluted in 1% BSA in the permeabilization solution: rabbit anti-Piezo1 (ab128245, Abcam, RRID:AB_11143245; 1:500 dilution), rabbit IgG isotype control (31235, Thermo Fisher Scientific, RRID:AB_243593) (Supplementary Figure 1), mouse anti-A2B5 (MA1-90445, Thermo Fisher Scientific, RRID:AB_1954783), mouse anti-APC (CC-1) (ab16794, Abcam, RRID:AB_443473), mouse anti-MBP (MA5-15922, Thermo Fisher Scientific, AB_11154789). The following day, the slices were washed twice with PBS for 10 min, then in the permeabilization solution once for 10 min and incubated for 2 h with secondary antibodies: anti-rabbit Alexa Fluor 488 (ab150077, Abcam, RRID:AB_2630356), anti-mouse Alexa Fluor 546 (A-11003, Thermo Fisher Scientific, RRID:AB_2534071). For the final rinsing, the sections were washed three times in PBS for 10 min and then once in distilled water. The sections were air dried, covered in Keiser’s gelatin and imaged using Zeiss 880 laser scanning confocal microscope (Zeiss, Germany).

### Cell Culture and Differentiation

MO3.13 human oligodendrocyte cell line (RRID:CVCL_D357) was purchased from Tebu-Bio (2018, CLU301, batch 131117 P25) and grown in high glucose DMEM supplemented with 10% fetal bovine serum (FBS) and 1% penicillin/streptomycin solution. To trigger maturation and differentiation, cells were treated with 100 nM PMA (P1585, Sigma-Aldrich) for 0–72 h or were grown in serum-free (SFM) media.

### Western Blotting

Sample proteins were extracted using RIPA buffer supplemented with protease inhibitor (Sigma, P8340) and kept frozen at −20°C until used. The samples were equalized using the BCA assay (23225, Thermo Fisher Scientific) and mixed with Laemmli buffer (1610747, Bio-Rad) supplemented with 5% β-mercaptoethanol and loaded in pre-cast gels (4561093, Bio-Rad) for PAGE-SDS electrophoresis. Proteins were transferred from the gel to a PVDF membrane using pre-assembled iBlot dry transfer stacks (IB401001, Thermo Fisher Scientific) in the iBlot gel transfer device (Thermo Fisher Scientific). After blocking with 5% non-fat milk in TBS with 0.5% Tween-20, membranes were incubated o/n at 4°C with the following primary antibodies: rabbit anti-PDGFRα (PA5-16571, Thermo Fisher Scientific, RRID:AB_10981626) and mouse anti-βactin (A5441, Sigma, AB_476744). Membranes were then washed and incubated with secondary antibodies: goat anti-rabbit IRDye 800 CW (925-68070, Li-Cor, AB_2651128) and goat anti-mouse IRDye 680RD (925-32221, Li-Cor, AB_621843), washed and let dry before being developed using an Odyssey Clx LI-COR Imaging system (LI-COR, NE, United States).

### Dot Blots

Primary human OPC lysate (ScienCell, 1606) prepared from early passage OPCs, primary human neuron lysate (ScienCell, 1526) prepared from early passage neurons and undifferentiated MO3.13 oligodendrocytes were used to quantify Piezo1 using dot blots. Two microliter of sample or 0.5 μL of rabbit anti-Piezo1 (ab128245, Abcam, RRID:AB_11143245) were spotted onto a nitrocellulose membrane. Non-specific sites were blocked by soaking the dried membrane in 5% milk in 0.05% tween-20 in PBS for 60 min at RT and then incubated o/n at 4°C with the primary antibody in the blocking solution. The membrane was washed 3 × 5 min with 0.05% tween-20 in PBS and incubated with secondary antibody conjugated to alkaline phosphatase for 60 min at RT, washed and incubated at RT in the dark in alkaline phosphatase buffer supplemented with NBT (Sigma-Aldrich, N6876) and BCIP (Sigma-Aldrich, B6149). The membrane was scanned with a standard office scanner.

### Quantitative Reverse Transcription PCR

Total RNA was extracted from frozen human brain sections. Visible periventricular plaques with adjacent healthy-appearing tissue was removed. Pieces of non-lesioned white matter were also selected and total RNA extracted. Human Oligodendrocyte Precursor Cell cDNA was purchased from ScienCell (1604) and Human Microglia Total RNA was purchased from Sti (37089RNA). All RNA was isolated from samples using the fenozol based Total RNA Mini Plus kit (#036-100, A&A Biotechnology) following the manufacturer’s guidelines. The quantity and quality of mRNA were measured by spectrophotometric analysis using a plate reader (Biotek, Synergy), RNA concentration was quantified using the optical density at 260 nm and samples were then equalized. The 260/280 ratios were used as a measure of quality and only samples with values between 1.8 and 2.0 were considered acceptable. cDNA synthesis was done using the TranScriba Kit (#4000-100, A&A Biotechnology) following the manufacturer’s guidelines and stored at 4°C until used. Quantitative reverse transcription PCR (RT-qPCR) was performed using TaqMan Master Mix (#4444556, Thermo Fisher Scientific) on the LightCycler480 (Roche, Switzerland). FAM dye-labeled TaqMan probes (Applied Biosystems, CA, United States) were used in all experiments. The relative mRNA expression using the ΔΔCt method was calculated from absolute quantification after normalization to the reference gene.

### Migration Assay

The migration assay was performed using the CytoSelect 8 μm-pore 24-well cell migration kit (CBA-100, Cell Biolabs). Briefly, 100,000 cells were seeded in the upper chamber of the transwell membrane in serum-free media with or without 500 nM GsMTx4 (#STG-100, Alomone Labs) or 40 μM Yoda-1 (#5586, Tocris), and in the lower chamber media containing 10% FBS was placed. Cells migrated for 24 h, after which the upper part of the membrane was cleaned with a cotton swab and then immersed in 100% methanol for 10 min. The transwell membrane was then washed and stained with crystal violet solution (V5265, Sigma) for 10 min. After staining, the transwells were washed and air-dried for imaging and subsequent incubation with 200 μL of extraction solution (methanol, included in the kit) for 10 min on a shaker. The solution was transferred to a 96-well plate and absorbance at 590 nm was read to quantify intensity using a Victor plate reader (PerkinElmer).

### MTT Assay

Cells were grown in a 24-well plate at a density of 120,000 cells/well. After treatments with GsMTx4 or Yoda-1, 0.5 mg/mL of 3-(4,5-dimethylthiazol-2-yl)-2,5-diphenyltetrazolium bromide (MTT; #M6494, Invitrogen) was added for 2 h. After incubation with MTT, 200 μL were removed and 150 μL of DMSO was added to solubilize the formazan. Then, absorbance was measured at 590 nm using a Victor plate reader (PerkinElmer).

### Live-Cell Calcium Imaging

Cells were seeded onto 24 mm culture glass coverslips (#1-6290, Bionovo) and grown o/n at 37°C in an incubator. Two hours before imaging, cells were incubated with 7.5 μM Cal-520 (ab171868, Abcam) in Hank’s balanced salt solution (HBSS, no phenol red) supplemented with 10 mM glucose and 25 mM HEPES for 90 min in the incubator and a further 30 min at room temperature, protected from light. Coverslips with dye-loaded cells were then washed once with HBSS without Ca^2+^ and Mg^2+^ and inserted into an AttoFluor acquisition chamber (Invitrogen). The cells were stimulated with either 20 μM Yoda-1 or HBSS for 40 s after 10 s of resting baseline. Time-lapse Ca^2+^ imaging was recorded with a Nikon Eclipse Ti fluorescent microscope (Nikon, Japan).

## Results

### Piezo1 Is Expressed in Oligodendrocyte Precursor Cells and Oligodendrocytes in the Human Brain

**FIGURE 1:**
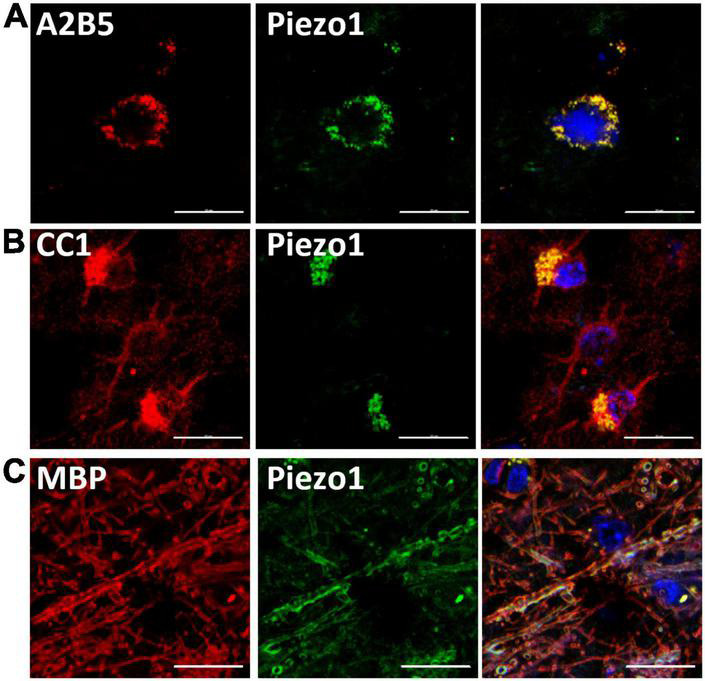
Piezo1 is expressed in oligodendrocyte linage cells in the human brain. **(A)** Piezo1 protein is present in OPCs (A2B5^+^), **(B)** in oligodendrocyte cell bodies (CC1^+^) and in **(C)** myelin (MBP^+^), as indicated by overlapping green (Piezo1) and red staining. Representative images of *N* = 3 human brains. Scale 20 μm.

First, we aimed to examine the presence of Piezo1 in the oligodendrocyte lineage cells in the human brain. We immunohistochemically stained brain sections and analyzed co-staining with anti-Piezo1 antibody and markers of oligodendrocyte linage cells: the OPC marker, A2B5, the oligodendrocyte body and membrane marker, CC1, and myelin basic protein (MBP), the myelin marker (Figure 1). We observed Piezo1 in OPCs (Figure 1A), oligodendrocyte cell body (Figure 1B) and mature myelin (Figure 1C). Piezo1 mRNA expression was also confirmed in primary human OPCs and microglia (Supplementary Figure 2A) and protein in primary human neurons and OPCs (Supplementary Figure 2B).

### Activation of Piezo1 With Yoda-1 Induces Calcium Waves in MO3.13 Oligodendrocytes

**FIGURE 2:**
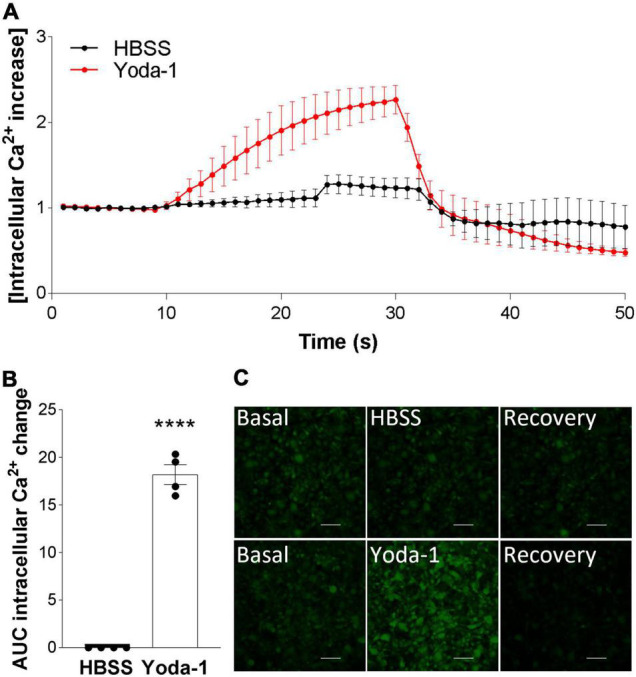
Piezo1 agonism with Yoda-1 induces calcium signaling in MO3.13 oligodendrocytes. **(A)** Traces calculated from 50 s recordings show changes in Ca^2+^ levels overtime after stimulation with Piezo1 agonist Yoda-1 (20 μM) and no increase after HBSS (ctrl). Peak from *t* = 10 s to *t* = 24 s, AUC = 18.19 ± 1.49. Traces are shown as mean ± SEM of *N* = 4 independent experiments. **(B)** Corresponding bar graph shows increased Ca^2+^ signaling after receptor stimulation with agonist Yoda-1. Data presented as mean ± SEM, unpaired *t*-test, ****p < 0.0001, *N* = 4 independent experiments. **(C)** Representative images taken from microscope recordings show basal levels of Ca^2+^, followed by induction of Ca^2+^ after addition of Yoda-1 or HBSS (ctrl), and subsequent recovery. Scale 100 μm.

After confirming Piezo1 presence in the human brain in OPCs and mature oligodendrocytes, we proceeded to investigate its signaling in these cells. The data showed that activation of Piezo1 with its agonist Yoda-1 led to the induction of Ca^2+^ waves in the MO3.13 oligodendrocytes (Figure 2). Recordings of live-cell Ca^2+^ waves with the Ca^2+^ probe Cal-520 showed that after stimulation with 20 μM Yoda-1, there was an increment in the intracellular Ca^2+^ load in the cells that lasted for *circa* 20 s and then declined to basal levels. This observed Ca^2+^ influx was not observed when the cells were only loaded with the HBSS buffer (control), indicating that the increase in Ca^2+^ signal after treatment with Yoda-1 was indeed induced by Piezo1 activation in these cells. Taken together, these data indicate that Piezo1 is present and functional in human MO3.13 oligodendrocytes.

### Inhibition of Piezo1 Increases MO3.13 Oligodendrocyte Migration *in vitro*

**FIGURE 3:**
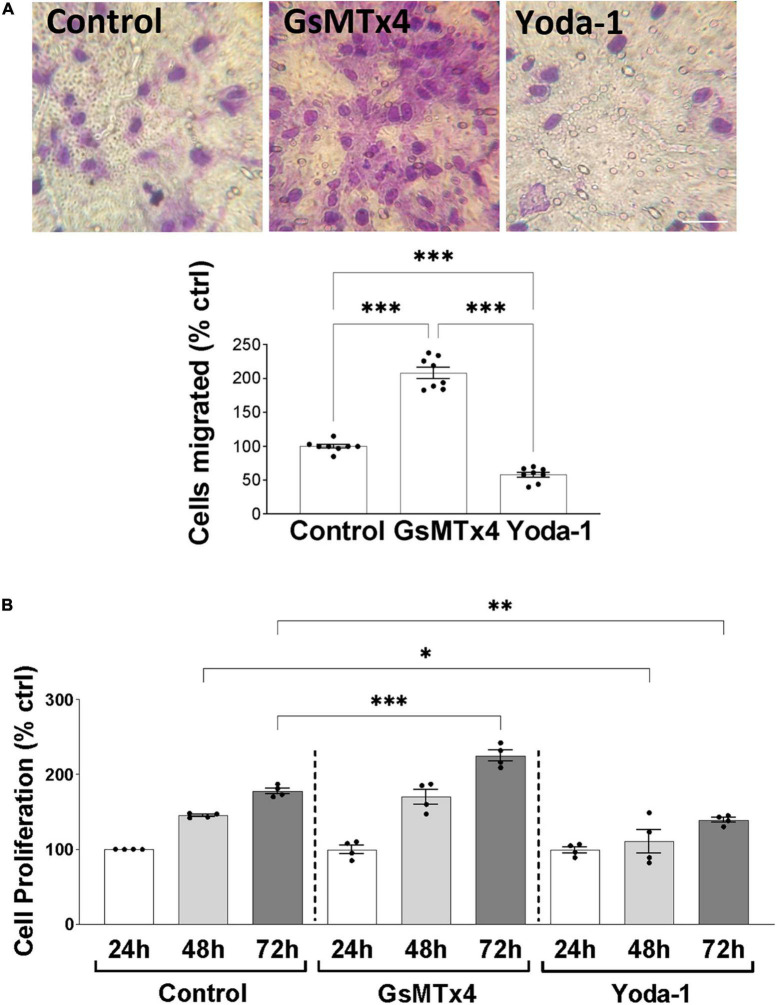
Inhibition of Piezo1 induces MO3.13 oligodendrocyte migration. **(A)** Representative images and a corresponding bar graph show increased oligodendrocyte migration after inhibition of Piezo1 signaling for 24 h with GsMTx4 (500 nm) (208.4 ± 8.21% vs. control). Agonism of Piezo1 with Yoda-1 (40 μm) induced opposite effects (58 ± 3.82% vs. control). Data presented as mean ± SEM, One-way analysis of variance and Tukey’s multiple comparisons test, ***p < 0.001, *N* = 8. Scale 25 μm. **(B)** The MTT cell proliferation assay showed significantly increased cell proliferation after 72 h of Piezo1 inhibition with GsMTx4 (224.8 ± 7.50% vs. control) with no differences compared to the 24 and 48 h controls. Activation of the receptor with Yoda-1 significantly reduced cell proliferation after 48 (110.8 ± 15.58% vs. control) and 72 h (139.0 ± 3.34% vs. control). Data presented as mean ± SEM, One-way analysis of variance and Sidak’s multiple comparisons test, *p < 0.05, **p < 0.01, ***p < 0.001, *N* = 4 independent experiments.

We continued our investigations of Piezo1 function in oligodendrocytes by examining the effects of Piezo1 signaling on cellular migration and proliferation, as these functions of OPCs are critical for proper myelination and remyelination in the CNS. Our data showed that inhibition of Piezo1 through GsMTx4 leads to an increase in cell migration to the lower chamber of the transwell assay (Figure 3A), compared to unstimulated cells (control). The activation of Piezo1 led to the opposite effect, causing a decrease in the number of cells that migrated, both compared to unstimulated cells and cells treated with GsMTx4. In addition to affecting cell migration, inhibition of Piezo1 signaling with GsMTx4 induced an increment in MO3.13 proliferation at 72 h compared to the unstimulated cells. On the other hand, Yoda-1 retained cell proliferation at lower rates at 48 and 72-h time-points compared to the unstimulated (control) cells (Figure 3B). These data show that increased Piezo1 signaling inhibits undifferentiated oligodendrocyte proliferation and migration.

### Expression of Piezo1 in MO3.13 Oligodendrocytes Decreases With Maturation

**FIGURE 4:**
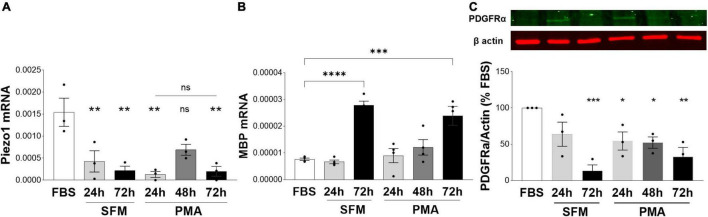
Expression of Piezo1 decreases in MO3.13 oligodendrocytes during maturation. **(A)** The mRNA expression of Piezo1 receptor in MO3.13 oligodendrocytes is downregulated already after 24 h of both, serum-free media (SFM) (28 ± 16% SFM 24 h vs. FBS) and SFM supplemented with PMA (8 ± 4% PMA 24 h vs. FBS). At 48 h of treatment with PMA *(data not collected for 48 h SFM)* Piezo1 mRNA briefly increases (45 ± 8% PMA 48 h vs. FBS, *p* = 0.0522) indicating a temporary upregulation of Piezo1 signaling during oligodendrocyte maturation. Data presented as mean ± SEM, One-way analysis of variance with Sidak’s multiple comparison’s test, *p* = 0.013, *N* = 3 independent experiments. **p < 0.01 **(B)** SFM (365 ± 20% SFM 72 h vs. FBS) and PMA (314 ± 46% OMA 72 h vs. FBS) treatment induce MO3.13 oligodendrocyty maturation. Data presented as mean ± SEM, One-way analysis of variance with Sidak’s multiple comparison’s test, *p* < 0.0001, *N* = 4 independent experiments. ***p < 0.001, ****p < 0.0001 **(C)** Representative WB image of OPC marker PDGFRα shows steadily decreasing levels of PDGFRα protein in MO3.13 oligodendrocytes stimulated with PMA or cultured in SFM. Data presented as mean ± SEM, One-way analysis of variance with Dunnett’s multiple comparison’s test, *p* < 0.0026, *N* = 3 independent experiments. *p < 0.05, **p < 0.01, ***p < 0.001.

As we observed an inhibitory role of Piezo1 signaling on proliferation and migration of oligodendrocytes, we next investigated whether Piezo1 expression changes with the maturation of MO3.13 oligodendrocytes. To do so, we induced the maturation of MO3.13 cells by two methods frequently used with this cell line, serum-starvation (serum-free media, SFM) (Haq et al., 2003; Issa et al., 2003) and treatment with phorbol 12-myristate 13-acetate (PMA) (Won et al., 2013; Guest et al., 2015). We found that Piezo1 expression significantly decreases already after 24 h in SFM or PMA. Interestingly, the expression of Piezo1 briefly increased after 48 h of PMA treatment (no data recorded for 48 h SFM) suggesting a temporary increase in Piezo1 signaling during oligodendrocyte maturation (Figure 4A). The functional outcome of this temporary increase in expression needs to be further explored. We corroborated the maturation of MO3.13 oligodendrocytes by measuring changes in the expression of the OPC marker platelet-derived growth factor receptor A (PDGFRα, decreases with maturation) and myelin protein, MBP (increases with maturation). The data showed that MBP mRNA levels significantly increase with maturation at 72 h in SFM and PMA (Figure 4B) and PDGFRα protein levels steadily decrease with time in SFM and PMA treatment (Figure 4C) confirming MO3.13 maturation and differentiation in our experiments.

### Piezo1 Is Downregulated in Multiple Sclerosis Brains

**FIGURE 5:**
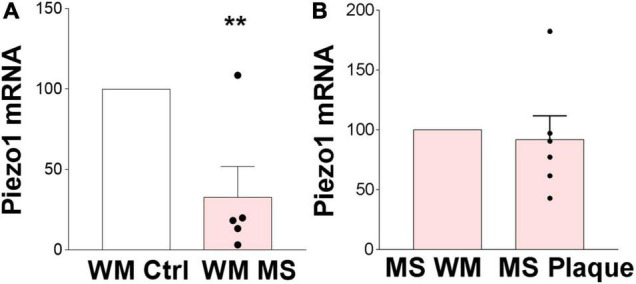
Piezo1 expression is downregulated in MS brain. **(A)** Analysis of Piezo1 mRNA levels shows significantly reduced expression of Piezo1 in MS brain in white matter (WM) (32.7 ± 19.2% vs. ctrl). Unpaired Student’s *t*-tests, **p < 0.005, *N* = 2 ctrl and *N* = 5 MS brains. **(B)** No differences in Piezo1 expression were found in the MS brains between WM lesions (plaques) and lesion-free WM within the same MS brain (91.98 ± 19.77% plaque vs. WM). Unpaired Student’s *t*-tests, *p* > 0.05, *N* = 5 MS brains.

We here demonstrated that Piezo1 is present in a healthy human brain, specifically in the oligodendrocyte lineage cells, and is functional in human undifferentiated MO3.13 oligodendrocytes. Identification of a receptor that modulates the proliferative and migratory function of oligodendrocytes has important implications in demyelinating diseases such as MS, where an increase in OPC proliferation and migration toward demyelinated areas is highly desirable to enhance remyelination. We have previously shown that inhibition of Piezo1 in the rodent brain protects from LPC-induced demyelination (Velasco-Estevez et al., 2020a) implicating that Piezo1 signaling is involved in oligodendrocyte biology and/or myelination in the CNS under pathophysiological conditions. Here, we proceeded to examine the expression of Piezo1 in MS brains. The data showed that Piezo1 mRNA levels significantly decrease in MS white matter (WM) compared to non-MS (control) WM (Figure 5A). Unexpectedly, no differences were observed in Piezo1 mRNA levels between the MS plaque and healthy-appearing WM within the same MS brain (Figure 5B) indicating that Piezo1 downregulation in the MS brain occurs at the whole-brain level and is not specific to demyelinated or degenerated brain areas.

## Discussion

Here we showed that the mechanoreceptor Piezo1 is present in human oligodendrocytes and OPCs in the CNS and that its expression is decreased in the white matter in the MS brain compared to non-MS white matter. Furthermore, our data showed that Piezo1 plays important role in oligodendrocyte biology and function. Activation of Piezo1 in the MO3.13 oligodendrocytes inhibited proliferation and migration, while the opposite effect, enhanced proliferation and cellular migration, were observed when Piezo1 was pharmacologically inhibited with GsMTx4.

Mechanical properties of the brain are sensed through mechanoreceptors—proteins that can respond to these physical cues, usually by the opening of the pore allowing a cation influx that leads to diverse responses in the cell. Piezo1 is one of the main mechanosensitive ion channel which expression and function were described so far. In the CNS, its expression has been shown in neurons (Wang et al., 2019; Roh et al., 2020), microglia (Liu et al., 2021) and astrocytes upon different activation states (Velasco-Estevez et al., 2018, 2020b). Here, we add to the list of Piezo1-expressing cells human oligodendrocytes and OPCs. More interestingly, our data suggest that Piezo1 could have a modulatory effect on proliferation, migration, and maturation of oligodendrocytes, as its inhibition with GsMTx4 triggered those processes and the opposite effects were observed upon Piezo1 activation with Yoda-1. If indeed true, modulation of Piezo1 signaling in oligodendrocytes could become a potential drug target for remyelinating therapies.

OPCs and oligodendrocytes are mechanosensitive cells and it is known that the physical properties of the ECM can regulate their survival, proliferation, migration, and maturation, although the biochemical processes involved are yet to be understood. Indeed, the OPC microniche stiffens with age, and this change in the elasticity of the matrix is responsible for their age-related loss of function in terms of proliferation, migration, and differentiation toward myelinating oligodendrocytes (Segel et al., 2019). This stiffening of the tissue is also observed in chronic demyelinating lesions (Urbanski et al., 2019) due to the appearance of glial scar and aberrant production of ECM components, such as fibronectin. An abnormal increase in stiffness of the tissue could thus explain the failure to remyelinate in the chronic demyelinating plaques. We here demonstrated that expression of Piezo1 is significantly decreased in the white matter in MS brains compared to the white matter in healthy brains. We cautiously speculate that the downregulated expression of Piezo1 in MS brains could be a feedback mechanism where cells downregulate Piezo1 in a gradually stiffening microenvironment to rescue their mechanosensing properties thereby increasing OPC proliferation and migration. However, the data on Piezo1 expression in MS brains here reported is purely descriptive and we cannot draw any conclusions relating to the function of Piezo1 in MS. Research in MS mouse models and further *in vitro* functional assays need to be conducted to understand the consequences or potential benefits of Piezo1 downregulation in the diseased brain.

## Data Availability Statement

The raw data supporting the conclusions of this article will be made available by the authors, without undue reservation.

## Ethics Statement

Post-mortem human brain sections were obtained from the Rocky Mountain MS Center Tissue Bank (Englewood, CO, United States) after approval by the bioethics committee at Medical University of Gdańsk, Poland (NKBBN/253/2018). Written informed consent for participation was not required for this study in accordance with the national legislation and the institutional requirements.

## Author Contributions

MV-E and AR contributed to the conception and design of the study, performed the statistical analysis, prepared the figures, and wrote the manuscript. MV-E and NK performed the *in vitro* experiments. AR secured the funding, performed the final staining, and analyzed the images. IK performed preliminary staining of the human brain (not shown). FC performed the mRNA analysis of the human brains. All authors read and approved the submitted version.

## Conflict of Interest

The authors declare that the research was conducted in the absence of any commercial or financial relationships that could be construed as a potential conflict of interest.

## Publisher’s Note

All claims expressed in this article are solely those of the authors and do not necessarily represent those of their affiliated organizations, or those of the publisher, the editors and the reviewers. Any product that may be evaluated in this article, or claim that may be made by its manufacturer, is not guaranteed or endorsed by the publisher.

## Supplementary Material

Supplementary Figure 1 Antibody specificity staining. Representative images of human brain sections immunohistochemically stained with rabbit IgG isotype control antibody for rabbit Piezo1 antibody. Scale 20 μm.

Supplementary Figure 2 Piezo1 is expressed in oligodendrocyte lineage cells. **(A)** Human primary OPCs express 50-fold higher mRNA levels of Piezo1 than primary human microglia. Data presented as average of technical duplicates of *N* = 1 lysates. **(B)** Dot blot shows Piezo1 protein levels in lysates of primary human neurons, OPCs and MO3.13 oligodendrocytes. Piezo1 protein is present in all three cell types with highest expression in MO3.13 oligodendrocytes. Bar graph shows densitometry quantification of dot blots (*N* = 1).
