# Supplementary figures and images for: Mechanoreceptor Piezo1 Is Downregulated in Multiple Sclerosis Brain and Is Involved in the Maturation and Migration of Oligodendrocytes in vitro
Source: Front Cell Neurosci. 2022 May 26;16:914985. doi: 10.3389/fncel.2022.914985 (PMC9204635; doi:10.3389/fncel.2022.914985)

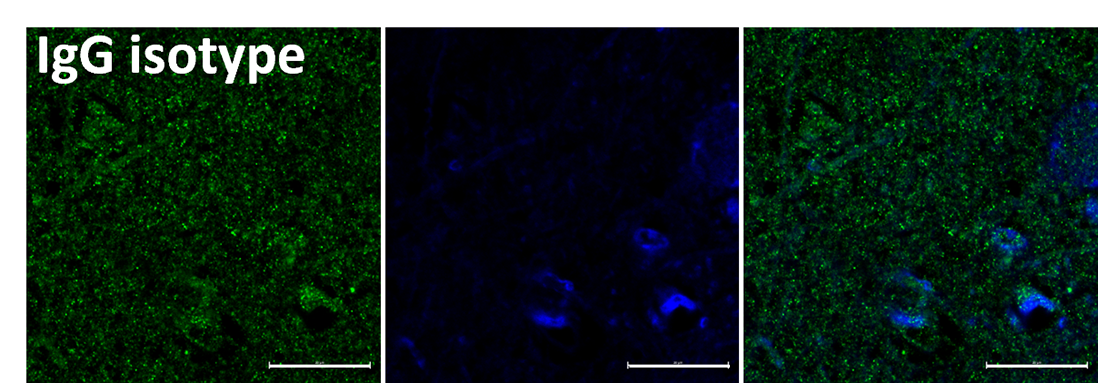

Supplement: Supplementary Figure 1 — Antibody specificity staining. Representative images of human brain sections immunohistochemically stained with rabbit IgG isotype control antibody for rabbit Piezo1 antibody. Scale 20 μm. [file Image_1.tif]

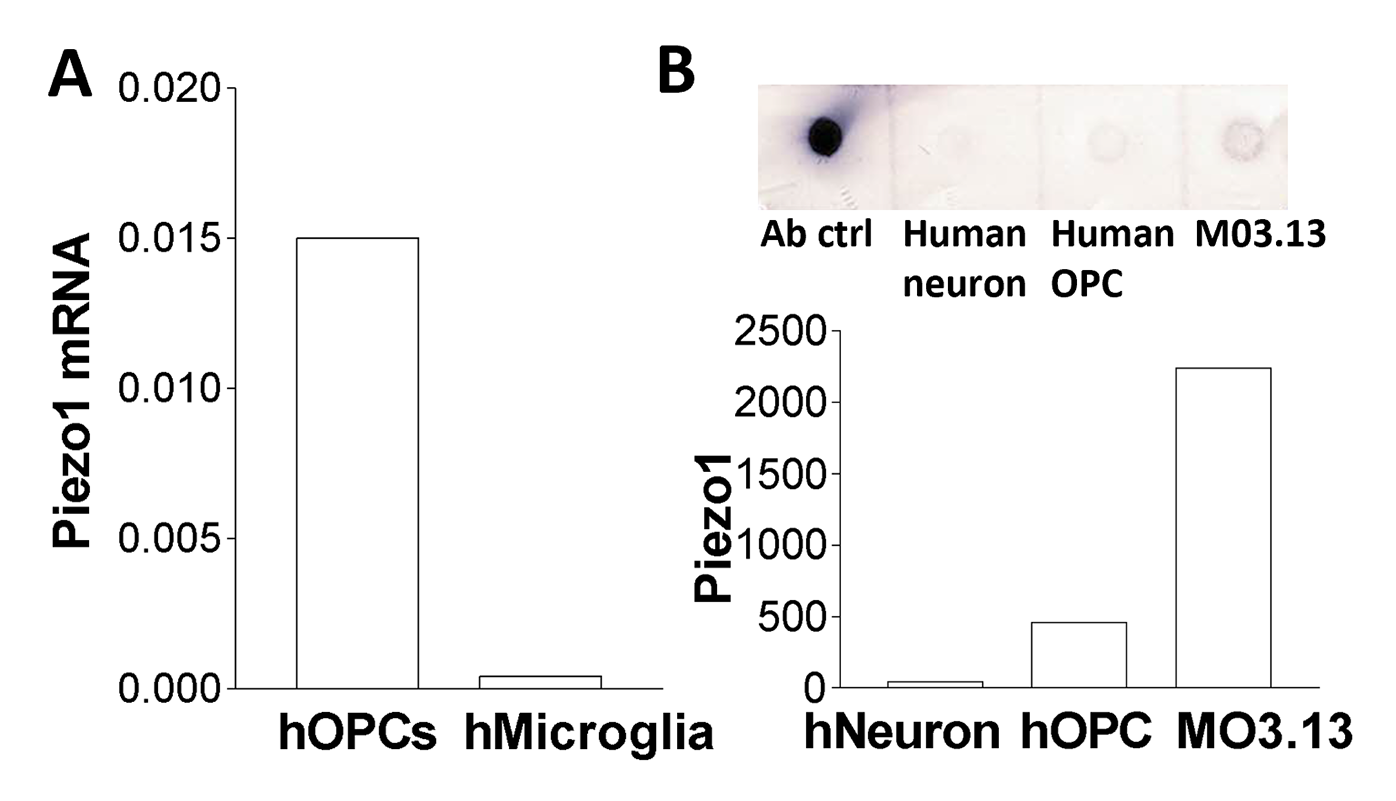

Supplement: Supplementary Figure 2 — Piezo1 is expressed in oligodendrocyte lineage cells. (A) Human primary OPCs express 50-fold higher mRNA levels of Piezo1 than primary human microglia. Data presented as average of technical duplicates of N = 1 lysates. (B) Dot blot shows Piezo1 protein levels in lysates of primary human neurons, OPCs and MO3.13 oligodendrocytes. Piezo1 protein is present in all three cell types with highest expression in MO3.13 oligodendrocytes. Bar graph shows densitometry quantification of dot blots (N = 1). [file Image_2.tif]
